# Drivers and barriers of AI tool adoption in Jordanian SMEs: a qualitative TOE-based study

**DOI:** 10.3389/frai.2026.1801583

**Published:** 2026-09-25

**Authors:** Hamad Alsawalqah, Yazn Alshamaila, Ons Al-Shamaileh, Ibrahim Aljarah, Ahmad Abadleh, Nailah Al-Madi

**Affiliations:** 1 King Abdullah II School of Information Technology, The University of Jordan, Amman, Jordan; 2 College of Interdisciplinary Studies, Zayed University, Dubai, United Arab Emirates; 3 Department of Computer Science, Mutah University, Al Karak, Jordan; 4 Computer Science Department, Princess Sumaya University for Technology, Amman, Jordan

**Keywords:** AI adoption, artificial intelligence, digital transformation, small and medium-sized enterprises (SMEs), technology organisation environment (TOE) framework

## Abstract

Artificial intelligence tools are increasingly used in business. However, small and medium-sized enterprises (SMEs) continue to face challenges in deciding whether to adopt them and how to. This study examines the factors that encourage or hinder the adoption of AI tools by SMEs in the Jordanian context. The study uses the technology–organisation–environment framework (TOE) and draws on 15 semi-structured interviews with AI solution providers, AI adopters, and firms considering adoption. The data was analysed thematically using TOE-guided deductive coding alongside inductive coding. The findings show that technological and organisational factors, including relative advantage, compatibility, managerial support, financial resources, and prior experience, influence AI adoption decisions. Environmental factors, such as competitive pressure, regulation, industry conditions, and public awareness, also affect adoption, but their role differs across firm categories. AI solution providers mainly emphasised expertise, market scope, and client readiness. Adopters focused more on organisational readiness and competitive pressure, while firms considering adoption reported uncertainty, cost, and employee acceptance as prominent barriers. Resistance to change and public awareness also appeared as context-sensitive factors within the organisational and environmental dimensions of TOE. The study provides qualitative evidence from Jordanian SMEs and offers practical implications for SMEs, AI solution providers, and support bodies in Jordan and similar regional contexts. The findings should be read as exploratory and context-specific, not as broad evidence for all SMEs in developing countries.

## 1 Introduction

According to the World Bank (2024), small and medium-sized enterprises (SMEs) make up over 90% of all companies and over 50% of global employment. In developing economies, SMEs provide up to 40% of the national income. The same is true in Jordan, where SMEs constitute around 98% of all firms (The Jordan Times, 2019). Therefore, promoting the growth of SMEs is a key strategy for advancing economic growth in the Middle East (Baabdullah et al., 2021; Lukonga, 2020). The promotion of company growth is linked to three primary factors: internationalisation, digitalisation, and sustainability. Specifically, the current economy emphasises the importance of digital transformation and artificial intelligence (AI) as key drivers of innovation and revitalisation of established small and medium-sized firms (Denicolai et al., 2021).

Recent statistics indicate that the utilisation of technology and AI may support the transition of around 75% of organisations to new projects and initiatives, while also empowering 84% of enterprises to obtain or maintain a competitive advantage. Thus, there is a growing desire among companies to adopt AI as part of the broader digital transformation (Hafnawi, 2021). As a result, the Jordanian government has implemented several steps to advance the digitalisation of industries, enterprises, and public services (Adaileh and Alshawawreh, 2021). Jordan is progressively moving towards integrating ICTs throughout the entire economy while emphasising specific markets and global value chains (Adaileh and Alshawawreh, 2021). However, this increased interest in technology has had an impact on SMEs. They have been pushed to implement complex growth strategies to improve their competitiveness and adapt to evolving technological and social conditions (De Marco et al., 2020; Falahat et al., 2020). Furthermore, SMEs continue to face challenges in terms of their preparedness and integration of new technologies for digitalisation, especially in developing countries (Schröder, 2016). SMEs in Jordan employ AI to a comparatively limited extent, despite the widespread adoption of IT in their day-to-day operations (Lutfi et al., 2020). Previous research suggests that this limited adoption may be associated with barriers to AI adoption and differences in firms’ readiness to integrate advanced technologies. This persists despite the existence of several theories explaining technology adoption (Chatterjee et al., 2021).

This challenge is not limited to Jordan but is also evident in neighbouring countries, such as the UAE, where SMEs contribute around 75% of the GDP and 95% of employment (Ravichandran and Krishnamoorthy, 2024). However, many of these enterprises still lack the ability to leverage AI technologies to improve their procurement processes, relying instead on traditional methods that are not data-driven, leading to missed opportunities for improvement (Al Shamsi, 2025). Moreover, in Saudi Arabia, the opportunities, obstacles, and factors affecting the success of new digital business models still receive limited attention from society (Tripathi and Singh, 2024). This points to a broader need for structured guidance to support enterprises in making AI adoption and procurement decisions (Al Shamsi, 2025).

Although technology adoption has been studied for many years, there are still certain gaps within this domain. For instance, Tornatzky et al. (1990) argue that the decision to adopt an innovation at the business level is impacted not only by technological variables but also by organisational and environmental settings. However, previous studies on technology adoption focused mainly on one TOE dimension. Even studies implementing the Technology-Organisation-Environment (TOE) theory were limited to a few fields and did not involve companies (Alsheibani et al., 2018), resulting in a limited exploration of the factors influencing companies’ decisions to employ AI (Chatterjee et al., 2021). Several studies started discussing the reasons for SMEs adopting AI solutions by using the TOE model, but the framework was often applied only partially. Rawashdeh et al. (2023) explored the technological factor influencing AI adoption in SMEs but did not include the organisational or environmental dimensions. Similarly, Chatterjee et al. (2021) integrated the technological and organisational factors through a combined TAM-TOE model, and offered a narrowed exploration of the environmental factors, such as competition and regulation. In contrast, Lada et al. (2023) offered an examination of the organisational and environmental factors, including top management and external support and omitting the technological dimension, whereas Nguyen et al. (2022) emphasised the technological and organisational factors without systematically examining the environmental factors. However, Baabdullah et al. (2021) put forth an examination related to antecedents of AI-based B2B practices without adopting a TOE-structured research design. Prior research in the MENA region has provided limited examination of all three TOE dimensions together in the context of SME AI adoption. Furthermore, it specifically targeted companies that depend on AI without considering the perspectives of companies that do not utilise these technologies or have plans to adopt them. In addition, most studies have examined the adoption of AI tools in developed countries, with limited attention given to the Middle East region, particularly Jordan.

To bridge this gap, this study examines the technological, organisational, and environmental determinants influencing AI adoption decisions among Jordanian SMEs, using the Technology-Organisational-Environment (TOE) framework. Unlike previous TOE-based studies that have largely focused on AI adoption from a single stakeholder perspective, this study adopts a multi-perspective qualitative design, integrating the views of AI solution providers, SMEs that have adopted AI tools, and SMEs considering adoption. This enables triangulation of adoption determinants from complementary stakeholder perspectives, reducing reliance on a single organisational viewpoint and providing a richer explanation of AI adoption decisions. A common analytical focus on adoption determinants was maintained across all three stakeholder groups. The study aims to contribute to the application of the TOE framework by exploring context-sensitive determinants and offering practical insights for SMEs, AI solution providers, and policymakers in emerging economies. The study addresses the following research question:

*RQ1*: What are the main factors influencing the propensity of SMEs to use AI tools?

The rest of this paper is organised as follows: Section 2 reviews the existing literature related to SMEs, AI tool adoption, and the TOE framework. Section 3 presents the research approach, which consists of qualitative analysis, and the method of selecting the target companies. Given the exploratory nature of the research question, aiming to identify the factors and challenges facing AI adoption, a qualitative approach was chosen. Furthermore, prior TOE-based studies relied on a similar approach to produce more accurate context-grounded insight, which is the case in Jordan given the small and heterogeneous population of SMEs adopting AI, limiting the feasibility of large-scale survey-based sampling. Section 4 presents the results followed by Section 5, which includes the discussion and implications of the study. The conclusion, limitations of the study and areas for future research are presented in Section 6.

## 2 Literature review

### 2.1 SMEs and AI tool adoption

AI tools have been used in a wide range of domains, and the world is witnessing an increase in the use of AI tools in many domains, including healthcare, education, industry, manufacturing etc (Celik, 2023; Ghobakhloo and Ching, 2019; Xu et al., 2019). According to a McKinsey and Company (2018) survey, the adoption of AI tools is spreading rapidly among global businesses. In the business domain, the development of SMEs is notable since it creates employment opportunities and enhances economic growth (Zou et al., 2021). SMEs are generally defined as independent businesses with fewer than 250 employees (European Union Commission, 2003). Recently, the digitalisation driven by the fourth industrial revolution has encouraged SMEs to use technological products (Ghobakhloo and Iranmanesh, 2021; Maroufkhani et al., 2023). This increases the adoption of smart systems and new technologies such as AI, internet of things (IoT) and blockchain (Hansen and Bøgh, 2021; Verma et al., 2021).

In this context, AI adoption offers a range of benefits for SMEs, which can increase competitive advantage (Mills, 2019). AI tools can improve decision-making by reducing costs, accelerating work, and improving company performance and sales (Borah et al., 2022). Moreover, AI adoption among SMEs restructures and enhances ordinary tasks such as marketing, customer service management and sales interactions with consumers and SEO (search engine optimization) (Sharma et al., 2022). AI can also contribute to the building of flexible and interactive solutions to customers’ needs (Strang and Sun, 2019). Likewise, a systematic review conducted by Lu et al. (2022) found that the benefits of AI adoption for SMEs are enhancing process efficiency, obtaining real-time customer insights, innovating products and services, reducing business risk and demand forecasting.

Accordingly, SMEs may use different AI applications to capture these benefits. AI applications such as deep learning, machine learning, and process automation are the main technologies used to achieve the desired benefits (Davenport, 2018). Generally, AI tools are divided into AI-based customer support tools, AI-based marketing tools, AI-based tracking and analytics tools, AI-based accounting and compliance tools, and AI-based cybersecurity tools. Prior literature shows that some technologies and tools are used in small firms (Basri, 2020; Ghobakhloo and Ching, 2019; Nadarzynski et al., 2019; Selamat and Windasari, 2021).

In their study, Baabdullah et al. (2021) reviewed the prior literature and examined adoption and implementation of AI by organisations. They found that these studies focus on antecedents rather than consequences of AI adoption. For instance, Basri (2020) examined the use of AI tools in social marketing by designing a questionnaire survey for SMEs in Saudi Arabia. They also found that AI tools improve business management and enhance SMEs performance. Similarly, in the manufacturing sector, Ghobakhloo and Ching (2019) designed a questionnaire survey for manufacturing SMEs in Iran (*n* = 183) and Malaysia (*n* = 177). Their results showed that strategic roadmapping for manufacturing digitalisation is the most significant determinant of SMIDT adoption. Another aspect of AI-based tools is chatbots. Chatbots offer a wide range of opportunities to organisations to enhance SMEs’ competitiveness and improve customer service (Sharma et al., 2022). In a study by Subagja et al. (2023), the use of ChatGPT as an AI tool in SMEs can help improve the quality of customer service.

On the other hand, prior literature has shown that SMEs still struggle with digitalisation in terms of readiness for and adoption of new technologies. Regardless of the diverse advantages of AI adoption, SMEs remain cautious about adopting digital solutions (Schröder, 2016). This is due to several barriers seen in the literature such as lack of strategy, governance model, constrained end-to-end solutions and competence, which are considered vital barriers when adopting AI solutions in SMEs (McKinsey and Company, 2018; Jabłońska and Pólkowski, 2017). McKinsey and Company (2018) states that only 18% of SMEs have a clear strategy to collect data and use AI. The literature reveals that the size of the organisation has a main role in adoption of AI. Large organisations can easily invest in IT budgets (Corea, 2017). Smaller organisations choose long-term AI research and development instead of short-term business applications due to the costs (Corea, 2017). Therefore, several studies have examined the adoption of new technologies by SMEs using different frameworks to identify the barriers that affect the adoption of AI tools (Ghobakhloo and Ching, 2019; Horváth and Szabó, 2019; Lu et al., 2022; Masood and Sonntag, 2020). In this regard, the TOE theory is widely used. This theory provides a broader view on the case. The following section provides more insights into the adopted theory.

### 2.2 Theoretical framework

#### 2.2.1 Technology–organisation–environment (TOE) framework

Several theories have been proposed in the literature to investigate the adoption of new technologies: Technology Acceptance Model (TAM) (Davis, 1989), Theory of Planned Behaviour (TPB) (Ajzen, 1991), Diffusion of Innovation (DOI) (Rogers, 1995), Unified Theory of Acceptance and Use of Technology (UTAUT) (Venkatesh et al., 2003). Unlike these theories, TOE includes three contexts to examine adoption.

The Technology-Organisation-Environment (TOE) framework was developed by Depietro et al. (1990). This framework describes businesses’ decision-making behaviour regarding technology innovation. This model attracts attention since it includes the main conditions needed to examine adoption. This model explains how the technological, organisational and environmental contexts influence the implementation of technology. In this regard, TOE is considered as a successful model to identify the factors that influence the implementation of emerging technologies by SMEs (Baker, 2011). The TOE model gives flexibility to the technological, organisational and environmental factors that influence the adoption process by the organisations. Including all contexts, it improves the ability to explain the factors (Hsu et al., 2014). The flexibility of this framework allows new dimensions that may affect the adoption decision as well as modification of the factors to fit emerging technologies. Prior literature shows that this model is used to distinguish between adopters and non-adopters (Sun et al., 2020). Overall, the TOE framework provides a useful lens to analyse the factors in the context of AI adoption.

The TOE framework provides a comprehensive approach to technology adoption and implementation in which the context-specific variables can be contextualised in the analysis of the challenges SMEs face when using AI tools in the Jordanian context. Regulatory and cultural factors can be investigated because TOE accounts for the external environmental impact. The TOE framework has been used widely in developing countries and specifically in the Jordanian context, e.g., cloud computing, big data, ERP systems, e-commerce etc (Rawash, 2021; Lutfi et al., 2022).

By applying the TOE framework, the study investigates the factors that influence the adoption decision. It further highlights strategic actions that help in overcoming these challenges.

The TOE framework consists of three contexts as follows:

*Technological factors* refer to the technological features that affect the businesses’ ability to adopt new technology.

*Organisational factors* refer to the organisational attributes that affect the businesses’ ability to adopt new technology.

*Environmental factors* refer to the external conditions around the firm and industry Table 1.

**Table 1:** Presents the TOE framework factors that influence the adoption of technologies.

TOE Dimension	Factor	Definition/Description
	Technological	Relative advantage	“The degree to which an innovation is perceived as being better than the idea it supersedes” (Rogers, 2003)

		Uncertainty	The extent to which the outcomes of using an innovation are uncertain (Fuchs, 2005).

Compatibility integration with existing systems alignment with business processes	“The degree to which innovation is perceived as consistent with the existing values, past experiences, and needs of potential adopters” (Rogers, 2003)

Complexity ease of use implementation effort	“The degree to which an innovation is perceived as relatively difficult to understand and use” (Rogers, 2003)
Organisational	Top management support	Devoting time to the [ICT] programme in proportion to its cost and potential, reviewing plans, following up on results and facilitating the management problems involved with integrating ICT with the management process of the business (Young and Jordan, 2008).
	Financial and human resources	SMEs with sufficient financial and human resources are more likely to adopt AI tools. Organisations need to invest in training and development to build internal capabilities.
Environmental	Competitive pressure	The degree of pressure felt by the firm from competitors within the industry (Oliveira and Martins, 2010).
	Industry	The sector to which the business belongs (Yap, 1990; Goode and Stevens, 2000).
	Market scope	The horizontal extent of a company’s operations (Zhu et al., 2003).
	Regulatory environment	Favourable regulations and policies, including data privacy laws and incentives for technology adoption, can encourage SMEs to adopt AI tools.

#### 2.2.2 Key TOE factors influencing the adoption of AI tools

Prior literature has shown that a number of studies have investigated the adoption of AI tools by SMEs using the TOE framework. These studies offer insights into how internal and external factors influence adoption decisions. Based on the review of the existing literature, this section investigates the factors most frequently reported in prior studies of SMEs AI adoption. This provides a conceptual foundation for the current study and helps identify the common determinants and the limitations in existing literature.

### 2.3 Technological factors

The technological factors refer to the technological features that affect the businesse’s ability to adopt new technology. In the context of SMEs, relative advantage, compatibility and complexity are identified as the key factors (Chakraborty et al., 2025; Ghobakhloo and Ching, 2019; Nguyen et al., 2022; Polisetty et al., 2024).

*Relative advantages:* Perceived advantages such as performance improvement, time saving and cost saving are influential when SMEs adopt AI tools (Apostoaie et al., 2025; Nguyen et al., 2022; Rawashdeh et al., 2023; Devkota et al., 2022; Chatterjee et al., 2022; Escobar et al., 2023). In their comprehensive study, Soomro et al. (2025) concluded that AI adoption provides sustainable performance for SMEs. Another study conducted by Abrokwah-Larbi and Awuku-Larbi (2024) shows that the performance of SMEs in Ghana has improved while adopting AI marketing tools. For SMEs, AI tool implementation offers a wide range of benefits such as reduced costs, better decision-making and enhancing performance (Badghish and Soomro, 2024).

*Compatibility:* Compatibility refers to the degree to which an innovation is aligned with the firm’s beliefs, existing values and experience (Rogers, 2003). In AI adoption, compatibility with the company’s beliefs and practices is found significant (Badghish and Soomro, 2024; Setiyadi et al., 2019; Chatterjee et al., 2021; Rawashdeh et al., 2023). When SMEs consider AI technology to be compatible with their needs and support their goals, they are likely to adopt it (Schwaeke et al., 2025). This implies that this factor plays a main role in shaping the adoption decisions.

*Complexity:* Technological complexity relates to the difficulties that the organisation faces in understanding, implementing and therefore using new technology. Regarding AI tool adoption, complexity is frequently identified as a major factor. Higher perceived complexity has a negative impact on the adoption of AI technologies (Chakraborty et al., 2025; Russo, 2024; Sharma, 2024; Susetyono et al., 2025). Sánchez et al. (2025) found that empirical studies have shown that SMEs prefer simpler AI solutions in specific fields related to operational efficiency and customer service automation. If the solutions offered by the AI tools are complicated, they are less likely to be implemented by the users (Sharma, 2024). However, in other studies related to developing countries, complexity has been found to be insignificant since the adoption of AI tools is still at early stages (Apostoaie et al., 2025; Badghish and Soomro, 2024).

### 2.4 Organisational factors

The organisational perspective refers to an organisation’s characteristics that impact its capacity to adopt and implement new technologies (Awa et al., 2016). Organisational capabilities encompass management, resources, and leadership in place to support adopting an innovation. These attributes are often specific to an organisation, cannot be transferred, and are deeply embedded inside the organisation (Nguyen et al., 2022).

*Top management support:* Top management support refers to the support given by upper management in adopting cutting-edge technology (Sharma et al., 2022; Sun et al., 2020). Daoud et al. (2021) state that top management commitment support is the extent of support and dedication exhibited by the management in an organisation towards a particular decision. According to Alsetoohy et al. (2019), A firm’s decision to adopt a new technology is favourably correlated with the motivation of top management to adopt the new technology. Studies have found that the support of managers is crucial for the adoption of technology (Nguyen et al., 2022). At the organisational level, top management support is among the top three crucial indicators for predicting the adoption of innovation (Lutfi, 2022). Therefore, in SMEs, top management support plays a crucial part in the process of making important strategic decisions (Lada et al., 2023). In the AI field, Baabdullah et al. (2021) investigated the previous studies related to AI adoption in B2B businesses. The results show that management support is an influential factor.

*Human Resources*: To capitalise on the opportunities presented by AI, companies have to develop the capabilities to effectively utilise AI technologies. The development of capabilities relies on human factors, including organisational leaders, employees, experts, and others (Lu et al., 2022). SMEs generally suffer from a shortage of human resources in comparison to larger organisations, which hurts their ability to innovate (Badghish and Soomro, 2024). Thus, companies are required to assume the financial responsibility of hiring consultants (Sharma et al., 2022). A significant shortage in expertise increases the complexity of managing AI systems which hinders AI implementation (Chakraborty et al., 2025).

*Financial support:* A business’ adoption of innovative technology is significantly influenced by the availability of resources (Sharma et al., 2022). Resource availability refers to the accessibility of resources that organisations can use to adopt new technology (Maduku et al., 2016). Consequently, the successful implementation of adoption relies on the presence of resources such as skilled workforce and financial capital (Polisetty et al., 2024). However, the adoption of technologies by SMEs that operate in several business sectors is an issue because they often need financial support (Fanelli, 2021; Oldemeyer et al., 2025; Susetyono et al., 2025), which constitutes the main difference between large and small-scale organisations (Baabdullah et al., 2021). Therefore, financial considerations are crucial when implementing new technologies as they often require significant investments in various areas, including technological infrastructure, data collection and management, skilled personnel recruitment, and ongoing system maintenance (Lada et al., 2023).

In addition to these main factors, recent studies have revealed that resistance to change is emerging as a context-sensitive factor in AI adoption in SMEs that can be classified within the organisational dimension of the TOE framework. Resistance to change encompasses the fear of job displacement, low digital competence and rigid hierarchies (Baabdullah et al., 2021; Oldemeyer et al., 2025; Sánchez et al., 2025; Susetyono et al., 2025). When it comes to technology adoption, Baabdullah et al. found that managerial resistance is a major factor in technology implementation. Similarly, Polisetty et al. (2024) reported that conventional leadership styles and practices hinder the AI adoption. However, resistance to change was not treated as a predefined construct in the initial TOE coding framework of the present study. Its relevance to the organisational context was subsequently considered in light of patterns emerging inductively from the interview data.

### 2.5 Environmental factors

When it comes to environmental readiness, several studies have investigated the external factors that affect the adoption decision such as competitive pressure, stakeholder pressure, government role and regulations (Jun et al., 2021; Badghish and Soomro, 2024).

*Competitive pressure:* One of the factors that SMEs have to take into consideration is market pressure. The need to keep pace with changing business models serves as a driver that motivates companies to improve their technologies (Wong et al., 2020). Prior studies have shown that competitive pressure is positively associated with the adoption decision of AI technologies (Apostoaie et al., 2025; Sharma, 2024; Sun et al., 2020). Chen et al. (2023) have stated that competitive pressure leads the companies to optimise AI technologies in order to improve customer service, increase the relative advantage and enhance the organisational performance.

*External support:* External support refers to the collaboration and the support provided by external entities to help the company implement the technologies to achieve their goals. Enhancing employee knowledge through support from the organisational partners facilitates the implementation of AI technologies (Asgari et al., 2017). This can be also achieved through the support of the technology providers in which both work together to achieve mutual goals (Nguyen et al., 2022).

*Regulatory environment:* Government support plays a crucial role in adoption of AI, especially for SMEs. This support can take several forms. An effective policy formulation can create a supportive environment for adoption (Boubaker et al., 2025). For SMEs, infrastructure funding, advisory support and targeted grants can effectively encourage the SMEs to invest and implement AI tools (Dey et al., 2024). However, regulatory constraints can significantly hinder the process of AI tool adoption and therefore discourage firms pursuing AI-driven innovation (Sánchez et al., 2025).

Alongside these factors, public awareness has been reported as a sub-factor that falls under the environmental dimension of the TOE framework. Public awareness refers to the understanding and the knowledge that the user has regarding AI tools’ capabilities, the benefits, and the risks. Prior studies have investigated managerial and employee awareness as a main factor that plays a role in AI adoption in SMEs (Baabdullah et al., 2021; Polisetty et al., 2024). Awareness also refers to the societal knowledge of AI technologies, their benefits, and the risks associated with their use.

Despite the growing use of TOE in AI adoption research, evidence from the Middle East, and particularly Jordan, remains limited. Existing studies have also varied in the TOE dimensions and constructs examined, with comparatively limited attention to how established TOE determinants operate alongside context-sensitive factors in SME AI adoption. The present study addresses this gap by examining technological, organisational, and environmental determinants of AI adoption among Jordanian SMEs while allowing additional context-sensitive factors to emerge from the interview data.

## 3 Methodology

### 3.1 Research design

A qualitative research design was adopted to explore the factors influencing AI adoption decisions among small and medium-sized enterprises (SMEs) through semi-structured interviews. Given the exploratory nature of the research objectives and the limited empirical understanding of AI adoption among Jordanian SMEs, the qualitative approach was deemed appropriate for extracting rich contextual insights. The study incorporates the perspectives of three complementary stakeholder groups: AI solution providers, SMEs that have adopted AI tools, and SMEs considering adoption. All participants were asked to consider the factors influencing AI adoption decisions, enabling researchers to examine AI adoption determinants from multiple stakeholder perspectives rather than a single organizational one. This approach also facilitated the identification of context-sensitive factors beyond those identified in previous studies.

The interview questions were constructed based on the TOE framework (Depietro et al., 1990). This framework is a comprehensive approach in which various factors can be examined. Semi-structured interviews were conducted to examine the technology, organisational, and environmental factors that could impact the decision-making process of SMEs in adopting AI tools. The interviews, across all three participant groups, focused on the decision-making process related to AI adoption (see Appendix, Questions Q2 and Q3). Accordingly, companies that had already adopted AI tools were asked to consider the factors that influenced their initial adoption decision, rather than focusing on their current operational use of these tools. Thus, the analysis integrates the perspectives of AI adopters, SMEs considering adoption, and AI solution providers to provide a deeper understanding of the determinants of AI adoption.

### 3.2 Data collection and analysis

In-depth, semi-structured interviews were conducted with 15 SMEs in Jordan from different sectors to ensure diversity and to gain rich and contextual insights into the drivers and barriers for adopting AI tools.

To ensure consistency across participant groups, all interview questions were designed to explore the factors influencing AI adoption decisions. Small and medium-sized enterprises (SMEs) that had already adopted AI tools were asked to reflect on the factors that influenced their initial adoption decision, rather than focusing on their implementation experiences. SMEs considering adoption discussed the factors shaping their current intentions, while AI solution providers described the factors, they typically observe influencing SME adoption decisions. This approach ensured that all participant groups contributed evidence related to the same analytical focus, enabling a meaningful comparison of stakeholder perspectives.

Interviews were conducted in English or Arabic according to participant preference. The interviews were recorded and transcribed, and Arabic excerpts were translated into English and the transcripts were reviewed to ensure accuracy. Participants were selected based on their experience as decision-makers and their direct involvement in the technological aspect. Each interview lasted approximately 40–45 min and was recorded using Zoom, transcribed and then analysed using thematic analysis. The interviews were analysed to explore patterns and themes related to the benefits, drivers and barriers associated with adopting and using AI tools.

The coding process was conducted by two researchers to ensure consistency and alignment with the aim of the study. Disagreements were resolved through discussion in coding interpretation. Thematic analysis was used to identify, analyse and interpret the patterns and themes (Clarke and Braun, 2014). The analysis involved familiarisation with the data; generating initial codes to identify meaningful concepts; constructing candidate themes by linking the categories to the technological, organisational, and environmental dimensions of the TOE framework; reviewing and refining themes; defining themes; and finally reporting the results (Clarke and Braun, 2014). First, the researchers repeatedly reviewed the transcripts and the recordings to achieve familiarity with the data. During this phase, initial notes were documented to support the coding process. Second, the researchers manually coded the data and text segments related to the research objectives were labeled. Deductive and inductive approaches were adopted to enable both theory-driven and data-driven analysis. First, initial coding was guided by the TOE framework (deductive coding). Then, additional themes that emerged from the data were identified through inductive coding. Third, conceptually related codes were grouped to construct potential themes (Ozuem et al., 2022) aligning with the TOE framework (e.g., relative advantage theme: the codes were efficiency gain and faster decision-making). Following that, the themes were reviewed by comparing them with the original transcripts to ensure that these themes reflect the participants’ experiences and to align with the research question. After that, the themes were refined and named within the TOE. Finally, the researchers began the final analysis by including representative quotations to illustrate each theme and support the findings. This process ensures both confirmation of existing TOE factors and identification of emerging themes from the obtained data. To ensure trustworthiness in this qualitative investigation, we employed the transcript verification, coded-data review, group comparison, and review of emerging themes and interpretations (Guba and Lincoln, 1994).

### 3.3 Sampling

Fifteen semi-structured interviews were conducted with participants occupying different roles in SMEs across various sectors, including healthcare, marketing, and consulting. Participants were selected to capture diverse stakeholder perspectives across sectors, and cases were identified through stakeholder recommendations and initial screening questions. Data collection continued until thematic saturation was reached. The final two interviews, which formed part of the total sample of 15 interviews, did not generate substantially new themes or insights and largely confirmed the patterns identified in the earlier interviews.

As shown in Table 2, the participants are referred to as F1, F2, F3… etc. The industry, Stakeholder group, the interviewee’s position and the size of the company are presented in the table.

**Table 2:** A summary of the SMEs’ background information.

No	Industry	Stakeholder group	Interviewee’s position	Size
F1	Healthcare	AI solution provider	Team member	Fewer than 10
F2	Content writing	Adopted	Founder	Fewer than 10
F3	Consulting and outsourcing	AI solution provider	Founder	Fewer than 10
F4	Construction	Adopted	Founder	14
F5	Consulting	Adopted	Team member/Architect	40
F6	E-commerce and marketplace	SME considering adoption	Founder	Fewer than 10
F7	Heavy industries	Considering the adoption of AI tools SME considering adoptionnot yet using them	Team member	25
F8	Fast food	SME considering adoption	Founder	150–180
F9	Telemedicine	AI solution provider	Founder	100
F10	Smart systems	Adopted	Manager	26
F11	Furniture industry	SME considering adoption	Founder	100–120
F12	health insurance	AI solution provider	Founder	25
F13	Detergent industry	SME considering adoption	Founder	100
F14	Software	AI solution provider	Founder	86
F15	Software	Adopted	Team member	200

## 4 Results

### 4.1 Factors influencing AI-tool adoption

The presented results are in line with the TOE framework, under the technological, organisational and environmental dimensions, highlighting the key factors affecting the adoption.

#### 4.1.1 Technological factors

The results show that relative advantage emerges as a salient factor in all stakeholder groups. Participants across the three stakeholder groups consistently associated greater perceived relative advantage with stronger willingness to adopt AI tools. Regardless of sectors and Stakeholder group, the results show that, if the tools improve performance, increase efficiency, improve user experience, and save time and effort, the participants are strongly in favors of the use of these tools.

#### 4.1.2 Relative advantage

Relative advantage appears to be a crucial factor that affects adoption. For the adopters’ group, AI tools presented a suitable solution to enhance performance and increase efficiency; this helps these SMEs to be leading competitors in their fields. However, SMEs considering the adoption are aware of the relative advantage of the AI tools, and this encourages them to adopt AI tools. For instance, several participants (F2, F5, F9, F12, and F14) explained that using AI tools helps them accomplish tasks faster than before since they can be done in minutes rather than hours. Other participants (F8, F9, F10, and F12) focused on the decision-making support in which AI tools offer better data-driven decisions in different aspects, such as marketing and customer service. Enhancing customer interaction is also reported to be a major advantage that encourages SMEs to implement AI tools. In the marketing field, for example, one of the participants stated, “generally speaking, the main benefit is that AI tools connect the buyer and the seller more effectively” (F6). Another participant stated, “AI tools today increase the efficiency, accuracy, and range of application as it can search for items on a larger scale and in different languages to increase the results and accuracy” (F14).

#### 4.1.3 Uncertainty

In comparison with the previous studies, it was found that AI tool adoption depends highly on the level of uncertainty (F7, F8, F11, and F13). The results reveal that this is more common in sectors where AI is not commonly used, such as manufacturing, and in industries that require great precision in output, such as engineering organizations. Participant (F11) justified their concerns by saying that the absence of local references or specialists who can educate factories on how to integrate AI creates uncertainty in determining if AI would be helpful in this sector. Additionally, one of the participants stated, “We do not have an advisory organisation that helps the factories in AI tool implementation” (F13). However, other participants expressed their high concerns about the accuracy of the results in the engineering field declaring, “We still have some concerns about the accuracy of the outcomes while using AI tools in our field” (F7). While participant F8 stated, “In the fast-food sector, AI tools are not accurate. When it comes to the call centres that receive the orders and handle the customers’ complaints, AI-driven customer service systems are not fully developed to capture the differences in the dialects spoken in the country.”

#### 4.1.4 Compatibility

Firms considering adoption face considerable difficulties since they use obsolete tools and software. New solutions provided by AI tools lack flexibility in this regard since they are incompatible with them, limiting their use of the tools. Participants (F12 and F13) assured that the outdated state of some factory equipment complicates integration of AI technologies. In the manufacturing sector, one of the participants stated, “AI implementation requires buying new machines or updating existing systems” (F13). However, participants (F4, F14, and F15) expressed their satisfaction about the level of compatibility using AI tools. On the other hand, F14 reported that while compatibility is a significant factor, it highly depends on the product and service offered using the AI tools.

#### 4.1.5 Complexity

The results show that complexity of AI tools is not a significant barrier for most firms considering the implementation process of the tools. The findings support the fact that participants have become more aware of the potential of AI technologies and are ready to learn. Participants (F2, F3, F10, F11, and F14) indicated that complexity is not a barrier if the value is considerable, as staff can be trained to use new tools. They certified thatas long as the use of AI tools will benefit their businesses, training is the solution. F14 reports that AI tools, on the contrary, made interaction with the tools easier and faster. On the other hand, some participants (F5, F6, F8 and F9) stated that within their domain complexity of some AI tools primarily requires technical expertise, and this leads to hesitation since these companies are still small. Participant (F8) stated, “The more complex the tools are, the need for training increases leading to higher costs.”

#### 4.1.6 Organisational factors

In this group, the analysis revealed that SMEs with sufficient financial and human resources are more inclined to adopt AI tools. Top management support also surfaced as a frequently cited factor. Participants emphasised the importance of leadership in decision-making and how it affects adoption. Prior experience was not reported by many adopters and potential adopters as a direct barrier to initial adoption, but AI solution providers viewed it as important for effective and cost-efficient implementation.

#### 4.1.7 Top management support

The results suggest that management support serves as a foundational factor for AI tool adoption. This coincides with the findings of Baabdullah et al. (2021) who reported top management support as a key factor. Participants (F2, F8, F11, and F14) recalled that the role of managers is crucial when it comes to adoption decision-making, as leaders who fostered the effective utilisation of these technologies, and committed to training were more likely to move from considering AI adoption to actually implementing the decision. Additionally, participants noted that limited managerial commitment at the stage of decision-making was likely hinder AI adoption and implementation. Participants (F8 and F11) emphasised that when the leaders understand the benefits of using AI tools and encourage their use, it facilitates adoption and leads to better overall performance. Notably, Participant (F14) observed that the next-generation leaders in family-business tend to display stronger support for AI adoption compared to the founder-generation management. This could emerge as a crucial factor in the Jordanian context as many of the SMEs in Jordan are family-owned businesses. This aligns with emerging family-business literature suggesting that generational involvement in leadership and management aids in increasing the firm’s openness to new technological transformation compared to the founder generation (Kammerlander and Ganter, 2015). Consequently, Soluk et al. (2026) found that generation transition in management is accompanied by a shift in attitudes regarding emerging technologies such as AI integration.

#### 4.1.8 Human resources and financial support

In accordance with previous studies (Oldemeyer et al., 2025; Polisetty et al., 2024; Sharma et al., 2022; Susetyono et al., 2025), financial support is important when adopting new technologies. The participants recalled that financial resources were a relevantly decisive factor when the adopting decision-making process was being effected given that these organisations anticipated the need for funds for the tools themselves and for training employees. “SMEs need a substantial budget to implement AI tools,” (F6). Participant (F8) also confirmed that financial resources are the main obstacle to utilizing AI tools. Participant (F14), as a provider, noted that many SMEs face financial problems when implementing AI, especially when new infrastructure is needed. The same participant also emphasised the need for human resources that are willing to train and implement these new tools. Contrary to previous studies (e.g., Badghish and Soomro, 2024; Chakraborty et al., 2025), the analysis showed that financial resources emerged as a prominent barrier in participants’ accounts, while human resources were not seen as a major barrier by several participants. Participant (F9), for instance, explained that with adequate training, employees can use AI tools effectively.

#### 4.1.9 Environmental factors

The analysis showed that external factors were frequently emphasised by participants when it comes to utilising AI tools. Among these factors, competitive pressure and government regulations were among the most emphasised. Participants consistently highlighted their critical role in adopting AI. Industry and market scope appear to be key factors. Participants noted that the industry to which the SME belongs is a strong motivator to utilise AI tools.

#### 4.1.10 Competitive pressure

The findings of the study are in sync with Apostoaie et al. (2025), Sharma (2024) and Sun et al. (2020). Participants noted that the actions of the competitors often lead them to explore and implement AI technologies. In a highly competitive market, SMEs confirmed that being up to date is not only about the advantages, but also about keeping pace with other competitors. Participant (F2) stated that when competitors use these technologies to enhance their business and succeed, it becomes critical for other businesses to implement AI solutions. Similarly, participant (F14) emphasised that many companies apply the after-sales service where most of the competitiveness is seen. Another participant (F4) added that competition is important. For example, if a Jordanian firm and a Chinese firm compete, the Jordanian company must endeavour to keep up to benefit from opportunities, even if the local market does not use AI tools. Implementing AI technologies gives the company a competitive advantage. As a result, it became a leading company in the market.

#### 4.1.11 Industry and market scope

The analysis of data obtained from several SMEs from different sectors revealed that the industry to which the SME belongs is significant. The result showed that SMEs from highly dynamic sectors, such as information technology and finance, are early adopters to gain advantage and to keep pace with other competitors (F1, F5, F6 and F10). However, the participants from sectors, i.e., manufacturing and engineering, are assured that because of the complex nature of their sector, the implementation of AI tools is not easy. SMEs in these sectors tend to see proven results before implementing AI tools (F7, F11 and F13). Regarding the market scope, it is only considered a crucial factor for organisations planning to expand internationally, as competition outside is more competitive. Participant (F3) stated that anyone who intends to expand their business widely must activate AI use. Additionally, participant (F14) emphasised that internationally active companies are faster at adopting AI tools as there is a need to unify policies and regulations among different branches.

#### 4.1.12 Regulatory environment

Government regulations play a significant role in using AI tools. The absence of regulations was highlighted by the participants as a main barrier. The analysis showed that the government and the industry regulations are considered as barriers in the Jordanian context. Clear regulations facilitate the use of AI tools by SMEs. Many participants confirmed that there are no clear regulations to ensure safety and ethical standards. Lack of regulations or unclear regulations leads to uncertainty and hesitation due to the lack of guidelines and standards. Participants (F7, F13, and F14) all agreed that lack of regulation increases the risks of adopting AI tools.

#### 4.1.13 Context-specific factors

The analysis of the interviews revealed two context-specific factors. These context-sensitive factors, alongside the existing factors in the TOE model, help comprehend how the internal and external factors affect the decision-making process. They are as follows:

#### 4.1.14 Resistance to change

The findings indicate resistance towards AI tool adoption among some employees and service users. This is consistent with the recent studies of Baabdullah et al. (2021); Oldemeyer et al. (2025); Sánchez et al. (2025); and Susetyono et al. (2025). This appears as a relevant factor that impels the implementation of AI tools. Resistance aligns with the organisational factors. Employees’ resistance is linked to the organisational attitudes and norms towards the usage of AI tools. It is embedded in the organisational culture. The potential replacement of employees by AI leads them to impede the adoption of these technologies and intentionally stop the firm from using them effectively, as the interviews revealed issues over employee adoption of AI. Certain employees perceive AI implementation as a danger. Several participants stated that employee resistance exists in our culture as well as the fear of replacement (F3, F4, F8, F9, F14 and F15). One of the participants confirmed this point by stating, “There are some concerns about losing jobs and career opportunities which may lead to resistance to AI adoption” (F3).

#### 4.1.15 Public awareness

Public awareness also emerged inductively from participants’ accounts and was subsequently mapped to the environmental context because participants associated it with external market and user perceptions surrounding AI. Participants described concerns among customers and organisations regarding AI capabilities, data use, and unfamiliarity with AI-enabled services. Several participants (F1, F3, F8, F10, F13, and F14) associated limited awareness with hesitation towards AI adoption or use. From the provider perspective, limited customer awareness was also described as affecting acceptance of AI-based solutions. Based on these accounts, public awareness was interpreted as a context-sensitive environmental factor rather than a predefined TOE construct.

The following table offers a synopsis of the context-specific factors and the affected groups Table 3.

**Table 3:** A summary of the context-specific factors.

Name	Participants reporting the factor	Stakeholder groups represented
Resistance	F3, F4, F8, F9, F14, and F15	AI tools provider Adopted Considering the adoption of AI tools in the future
Public awareness	F1, F3, F8, F10, F13, and F14	AI tools provider Adopted Considering the adoption of AI tools in the future

### 4.2 Barriers faced by SMEs in the implementation of AI tools

#### 4.2.1 AI solution provider

A major challenge is the data required for developing an adaptive model, as obtaining data without direct collection is difficult due to inadequate archives or users’ disinterest in providing input, making information gathering challenging. Moreover, the data used in AI require careful filtering and analysis prior to their input into models. Training AI models necessitates large computing resources, posing a problem for startups. Moreover, ongoing validation of model performance is crucial to avert data drift problems that compromise prediction accuracy.

#### 4.2.2 Adopted AI tools

One of the challenges that this group faces is the necessity of staying up-to-updated to effectively use AI tools and avoid issues. The infrastructure is insufficient as it fails to keep up with AI tools. Consequently, collaboration with third parties is necessary, as the development of this technology demands an appropriate infrastructure. Moreover, utilising these tools is complicated for the company’s employees, requiring them to research and understand these tools before application. They do not engage with this technology directly due to its uncertainty. However, awareness is present. For instance, firms recognise that the solution to the issue is using AI; nevertheless, the implementation is challenging because of the shortage in knowledge, digital infrastructure, and the personnel proficient in these technologies.

#### 4.2.3 Considering the adoption of AI tools in the future

Participants from SMEs considering AI adoption reported challenges associated with limited familiarity with AI applications in their industries, particularly in manufacturing and engineering-related contexts. The varied backgrounds of staff influence their use of AI tools, such as the staff who have not completed their education. In the engineering sector, too, confusion remains over the most effective utilisation of AI tools. Also, many companies use old software and machinery, which impairs their ability to integrate with current AI solutions. These accounts reflected uncertainty about how AI could be applied within existing organisational and technological conditions rather than experience with implementation itself.

## 5 Discussion and implications

### 5.1 Discussion

The interviews were conducted with SMEs (*N* = 15), categorised into three groups: AI solution providers (F1, F3, F9, F12 and F14), AI adopters (F2, F4, F5, F10 and F15), and SMEs planning to adopt AI in the future (F6, F7, F8, F11 and F13). The three groups were examined to compare how the same TOE factors were perceived across stakeholder perspectives.

The interviews suggest a diverse pattern of influencing factors based on the companies’ position on AI solution providers (F1, F3, F9, F12 and F14) demonstrated the highest readiness from a technological and organisational perspective, clearly distinguishing themselves in factors, such as relative advantage, compatibility, and managerial support, due to their technical expertise and reliance on competition and market demand as a primary driver.

AI adopters (F2, F4, F5, F10 and F15) recalled that technological considerations were relevant to their initial adoption decisions, but their accounts also placed considerable emphasis on organisational factors, particularly management support and resource availability. Some participants nevertheless reported continued uncertainty regarding how AI tools could be applied effectively.

In contrast, potential adopters (F6, F7, F8, F11 and F13) demonstrated more greater uncertainty in technological and organisational factors, particularly in terms of the possibility of experimentation or observing benefits. This is due to firms that want to employ AI in the future but are unsure how to apply for it. They are concerned about high prices, the unknown benefits of AI tools, as well as the company’s readiness and employee acceptance. As a result, top management support is critical to encouraging the use of AI tools.

These findings suggest that potential adopters remain constrained primarily by technological and organisational readiness, while environmental pressures and incentives may also influence their progression towards adoption.

The impact of technological, organisational, and environmental factors on the preparation of SMEs to implement AI tools varies according to the company’s classification as a provider, adopter, or potential adopter. For example, environmental pressure, such as competitive pressure and industry, appears less pronounced among AI solution providers compared to the other categories, as AI service providers often start because the service is absent in the market, which reduces competitive pressure. In other sectors, the use of AI tools arises from the competitive pressure among enterprises to enhance their services. The organisation’s readiness also differs between categories. For instance, as a service provider, there is higher preparedness due to the necessity of being experienced in AI. Consequently, it seems to be a less significant factor in comparison to enterprises seeking to implement solutions using AI techniques. Similarly, the top management support is less important for AI solution providers because they are already supportive of the use of AI.

Some factors did not appear closely tied to the company category. Industry-specific barriers were highlighted by the participants (F11) and (F13), who explained that the lack of AI prevalence in their field makes the adoption process more difficult. The same applies to the participants (F5) and (F7), whose work in engineering sectors limits the availability of accurate tools to support the adoption process. The Market Scope factor only appears to have an impact on companies seeking to expand abroad or collaborate with foreign companies, making it more relevant to strategies geared towards international markets. For that, we can see that this factor appears in the provider, the adopter, and the firms that intend to adopt.

Participants from adopting and potential adopters categories generally did not refer to the absence of prior AI expertise as a hindrance to adopting AI technology, as many conveyed a readiness to learn. However, when questioned about the lack of implementation, it was suggested that the primary difficulty stems from uncertainty regarding the most beneficial applications and their successful use, highlighting an actual need for prior experience to achieve improved solutions. By contrast, AI solution providers believe that prior experience is essential as it facilitates the most efficient and cost-effective implementation of AI.

The interviews, when recalling and anticipating their initial adoption decision, revealed that technological and organisational factors generally were more prominent in participants’ accounts than environmental factors on AI adoption. This pattern reflects that most participants, across the adopted and considering adopting groups, described having more control over technical and organisational decisions, such as management system adoption or internal training, compared to environmental factors outside of the company’s direct control. Notably, participants indicated that environmental factors become more important when companies intend to expand or enter foreign markets, when competitive and regulatory pressures become more apparent, as raised by many participants. Meanwhile, building technological and organisational readiness remains a priority in early adoption decisions.

Consequently, the key factors shaping the adoption decision of SMEs to use AI tools (in a retrospective form by adopted firms, and anticipated perspective of firms considering adoption), classified by company status, are summarised in Table 4.

**Table 4:** Key factors associated with AI adoption decisions across stakeholder groups.

Factor category	AI solution provider	Adopted SME	SME considering adoption
Main factors	Relative advantage, Prior experience, Market scope	Relative advantage, Complexity, Compatibility, Management support, Organisational readiness, Competitive pressure	Relative advantage, Compatibility, Complexity, Uncertainty, Management support, Organisational readiness, Competitive pressure, Industry

The results indicate that the greater the understanding of AI applications and tools and their integration into the organisation, the fewer the challenges and factors that affect the company. Consequently, firms that provide AI tools encounter fewer challenges due to their deep expertise with AI, whereas those considering its adoption face numerous challenges, such as their uncertainty regarding the utilisation of this technology and their limited familiarity with it.

Researching AI tools for businesses is crucial due to the valuable insights for developing effective strategies and interventions that facilitate successful AI adoption within enterprises. Numerous studies have offered strategies at both the local and regional levels, and upon further research, three main themes have emerged: the role of relative advantage, the influence of human-related variables, and the treatment of environmental factors.

Consistent with prior regional studies, this paper demonstrated that relative advantage is a dominant technological factor driving AI adoption. Almashawreh et al. (2024) showed that employees’ technical knowledge, technological infrastructure, and organisational support play a pivotal role in AI adoption in Amman’s SMEs. Although these factors hold significance, this research indicates that relative advantage is a pivotal pillar in AI implementation. When available, firms may actively consider the adoption of the tool, despite potential challenges or the necessity for learning, which is also confirmed by Abaddi (2025). The consistency of these factors across three diverse studies related to Jordanian SMEs suggests that relative advantage appears to be an important indicator of AI adoption in this market.

However, environmental considerations were not examined in Almashawreh et al. (2024) and Abaddi (2025) studies, which are especially crucial for companies aiming to expand internationally. Our findings suggest that the omission of such factors may undervalue a factor that is context-dependent rather than universally negligible, such as competitive pressure and industrial conditions, thus affecting firms providing AI solutions or planning to broaden their scope. Participants’ accounts suggest that environmental factors may become more salient for firms operating in broader or more competitive markets, where regulatory and market pressures are more pronounced. This indicates that the relevance of environmental factors in TOE-based SME research may depend partly on a firm’s market orientation and should therefore be interpreted in relation to its operating context.

Another point of comparison is the demographic and human-related variables. Almashawreh et al. (2024) found that demographic factors had no material impact on the adoption of AI. While our study did not explicitly examine demographic factors, some interviews suggested that the education level of employees and management age may affect adoption. Some interviewees indicated that in specific industries, such as furniture or restaurants, employees who have not completed their education find it more daunting to utilise these tools in comparison to others. Similarly, some accounts indicated that management age may influence adoption. This can also be linked to studies, such as Kammerlander and Ganter (2015) and Soluk et al. (2026), which researches younger management leaders, especially in family businesses, and their adaptability to new technological adoption. Although the theoretical model primarily focused on technological, organisational, and environmental factors, employee education and management age appeared as possible additional influences on AI adoption. These observations were limited and should be treated as exploratory, requiring further systematic investigation in future studies.

Numerous existing studies investigate the factors that influence business adoption, primarily from the perspective of firms that have yet to implement AI (Chatterjee et al., 2021; Lada et al., 2023; Nguyen et al., 2022; Baabdullah et al., 2021; Lu et al., 2022). However, organisations that have started adopting AI faced a different set of challenges once the implementation phase was under application, which were not the main aim of pre-adoption-focused studies. This distinction is observed in our findings, as firms that have already adopted AI tools, when recalling factors that initially affected their decision to adopt, emphasized that organizational factors, especially management support and resource availability, were decisive factors to enable the decision. On the other hand, firms still considering AI adoption emphasize that technological factors, especially relative advantage and compatibility, are conditions they are currently weighing.

Finally, this paper found that the application of ICT/AI tools by SMEs was not hindered by lack of prior technical experience, as observed by Asare et al. (2012) and Baabdullah et al. (2021), as firms are not primarily concerned about the ease and time required to build AI competence. This research highlights this issue, with numerous interviewees claiming that a deficiency in technical knowledge was not a concern for them and expressing a willingness to learn. However, when asked about their failure to implement these practices, some responses suggested that it came from uncertainty regarding which applications would be advantageous or a lack of clarity on how to utilise them effectively. Nonetheless, it is noticeable that many AI solution providers emphasise the significance of prior experience. This is due to their broad backgrounds in AI, which enable them to understand that AI can be used most cost-effectively, effortlessly, and with optimal performance when experience is available. For that reason, prior experience appears important for effective implementation.

### 5.2 Theoretical and practical implications

The contribution of this study extends beyond applying the Technology–Organisation–Environment (TOE) framework to a new empirical setting. Rather than examining AI adoption solely from the perspective of a single stakeholder group, this study adopts a multi-perspective qualitative approach that integrates the views of AI solution providers, SMEs that have adopted AI tools, and SMEs considering adoption. By triangulating these complementary stakeholder perspectives, the study provides a richer understanding of the technological, organisational, and environmental determinants influencing AI adoption decisions among SMEs. Furthermore, the findings refine the application of the TOE framework by demonstrating how established TOE determinants, together with context-sensitive factors emerging from the Jordanian SME environment, shape AI adoption decisions across different stakeholder perspectives. In doing so, the study contributes to the growing AI adoption literature by providing both theoretically grounded insights and practical guidance for researchers, practitioners, and policymakers seeking to support AI adoption in SMEs within emerging economies.

The findings of the research provide multiple implications for the research community, managers and AI solution providers. Theoretically, the findings of the paper add to the literature in several ways. First, the existing literature regarding the investigation of AI tool adoption in SMEs, especially in the Middle East remains relatively limited. The study addresses the literature gap regarding the limited number of studies that investigate the adoption of AI tools in SMEs, and the main factors that affect the adoption since SMEs play an evident role in economic growth. Therefore, the study provides qualitative evidence on factors that affect AI adoption in Jordanian SMEs. Second, the study refines the application of the TOE framework by examining AI adoption through complementary stakeholder perspectives rather than relying solely on a single organisational viewpoint. The results show that established technological, organisational, and environmental determinants remain relevant, while also revealing how their relative importance is shaped within the context of Jordanian SMEs. This provides a context-grounded basis for future comparative research across different organisational and national settings. Third, the study sheds light on context-specific factors, i.e., resistance to change and public awareness. These context-specific factors provide avenues for further theoretical investigation in future AI adoption research. For instance, the specific context aspects could be further investigated in future research.

Practically, the study provides valuable insights and recommendations for managers and decision-makers. First, managers should invest in training programmes to equip employees with the digital literacy and AI-related competencies needed to use AI tools effectively. The study also suggests that managers should foster an organisational environment that encourages employees to experiment with AI tools to enhance productivity. Because resistance to change emerged as a key organisational barrier, managers should adopt phased AI implementation strategies supported by employee engagement and continuous communication. Such an approach promotes clear communication, encourages employee participation, and helps reduce resistance to AI adoption.

For policymakers, the findings provide useful insights that may assist in designing policies to support AI adoption among Jordanian SMEs. Policymakers should promote national AI awareness initiatives targeting SMEs to improve understanding of AI opportunities and reduce misconceptions surrounding AI adoption. In addition, advisory organisations can support SMEs across different sectors by providing technical assistance, implementation guidance, and practical recommendations for adoption of AI. Concerns about regulatory uncertainty further underscore the value of clearer, more accessible guidance on AI-related requirements, particularly in areas such as data use, safety, and responsible implementation. SMEs operating in or planning to enter international markets should also pay closer attention to external factors, including competitive conditions and applicable regulatory requirements, when evaluating AI adoption.

## 6 Future directions and conclusion

This paper offers a deeper understanding of the technological, organisational, and environmental factors that affect AI tools adoption in Jordanian SMEs. The findings highlight that relative advantage consistently emerge as a prominent determinant across the three stakeholder groups, reflecting the importance of efficiency, performance improvement, and competitiveness. While organisational readiness and top management support were identified as important for adoption, factors, such as uncertainty and compatibility, continue to hinder firms still considering AI adoption. Importantly, the study suggests that Internal readiness—including technological infrastructure, resources, and leadership support—was generally more prominent in participants’ accounts of adoption decisions, while environmental factors appeared more salient in accounts involving broader or internationally competitive markets. Moreover, two context-specific factors, such as resistance and public awareness, emerged inductively as context-sensitive factors and were subsequently interpreted within the organisational and environmental contexts of TOE, respectively. Overall, the research suggests that building internal capabilities, supported by clear government regulations and awareness initiatives, may facilitate AI adoption in SMEs. By handling these internal and external challenges, Jordanian SMEs may better position to adopt AI and enhance their long-term growth and competitiveness.

### 6.1 Limitations and future research

Our study offers new insight into AI adoption among SMEs in Jordan. This focus provides a more in-depth context in an underexamined setting, but the findings are directly applicable mainly to similar institutional environments. Thus, extending this work to other countries would help broaden the applicability of the identified patterns. The cross-sectional qualitative design does not permit statistical testing of relationships among the identified factors. In addition, although participants were selected for their relevance to organisational or AI-related adoption decisions, the study did not systematically capture the perspectives of all organisational stakeholders, particularly employees. Observations concerning employee education and management age were limited and were not treated as formal constructs; these therefore require systematic examination in future research. Although the sampling across the three stakeholder groups offered a balanced sample, as there were five AI solution providers; five adopters, and five potential adopters; and the multi-sector coverage offered varied and meaningful insights, expanding the number of participants in future studies across sectors and stages would provide more fine-grained and sector-specific results. Finally, the study focuses on a fast-moving technological implementation, so the findings reflect a specific point in time and longitudinal research would add valuable insight on the development of AI adoption patterns and organisational familiarity with them. Building on this, future research can focus on applying a wider quantitative sampling and data analysis, implementing a longitudinal and multi-stakeholder design approach, and can examine specific industries and their adoption of AI to further enrich understanding of similar novel technological implementations among SMEs.

## Data Availability

The original contributions presented in the study are included in the article/Supplementary material, further inquiries can be directed to the corresponding author.
