# Supplementary material for: Drivers and barriers of AI tool adoption in Jordanian SMEs: a qualitative TOE-based study
Source: Front Artif Intell. 2026 Sep 25;9:1801583. doi: 10.3389/frai.2026.1801583 (PMC13649747; doi:10.3389/frai.2026.1801583)
Supplement: Supplementary file 1 [file Supplementary_file_1.docx]

**Supplementary Appendix 1. Semi-Structured Interview Protocol**

(1) Could you kindly provide us with an overview of your company's history? (Years since establishment/ main services industry/ Number of employees).

(2) What are the primary challenges/benefits of implementing AI tools in your company?

(3) Why has your company decided to use or not to use AI tools?

Level of AI adoption and Use in the company:

**(4)** What AI tools has your company adopted?

**(5)** To what extent do you suppose your company is aware of AI tools?

The impact of Technological, Organisational, and Environmental factors on the adoption of Artificial Intelligence tools:

If we categorise these facets into three areas (Technological, Organisational, and Environmental):

**(6)** Which technological factors do you suppose could influence the adoption of AI Tools in your company? For what reason?

**(7)** How do complexity, compatibility, relative advantage, and cost impact the adoption of AI Tools?

**(8)** Which organisational factors do you suppose could influence the adoption of AI Tools in your company? For what reason?

**(9)** How do top management support, human resources/prior knowledge, and financial support impact the adoption of AI tools?

**(10)** Which environmental factors do you suppose could influence the adoption of AI Tools in your company? For what reason?

**(11)** How do competitive pressure and external support impact the adoption of AI tools?
