# Supplementary material for: Drivers and barriers of AI tool adoption in Jordanian SMEs: a qualitative TOE-based study
Source: Front Artif Intell. 2026 Sep 25;9:1801583. doi: 10.3389/frai.2026.1801583 (PMC13649747; doi:10.3389/frai.2026.1801583)
Supplement: Supplementary file 2 [file Supplementary_file_2.docx]

Supplementary Appendix 2. Traceability Thematic Matrix

| **TOE Dimension** | **Construct (Table 1)** | **Interview question(s) (Appendix)** | **Representative raw code / quote** | **Final theme (Section 4)** |
| --- | --- | --- | --- | --- |
| Technological | Relative advantage | Q6, Q7 | F14: AI tools increase efficiency, accuracy, and range of application | Relative advantage (4.1.1) |
|  | Uncertainty | Q6, Q7 | F13: "We do not have an advisory organisation that helps the factories in AI tool implementation"; F7: concerns about accuracy of outcomes in engineering | Uncertainty (4.1.1) |
|  | Compatibility | Q6, Q7 | F13: "AI implementation requires buying new machines or updating existing systems" | Compatibility (4.1.1) |
|  | Complexity | Q6, Q7 | F8: "The more complex the tools are, the need for training increases leading to higher costs" | Complexity (4.1.1) |
| Organisational | Top management support | Q8, Q9 | F8, F11: leaders who understand AI benefits and encourage use facilitate adoption; F14: generational differences in leadership support | Top management support (4.1.2) |
|  | Financial and human resources | Q8, Q9 | F6: "SMEs need a substantial budget to implement AI tools"; F9: with adequate training, employees can use AI tools effectively | Human resources and financial support (4.1.2) |
| Environmental | Competitive pressure | Q10, Q11 | F2: competitors succeeding with AI makes adoption critical; F4: competing internationally requires keeping pace | Competitive pressure (4.1.3) |
|  | Industry | Q10, Q11 | F1, F5, F6, F10: dynamic sectors (IT, finance) are early adopters; F7, F11, F13: complex sectors (manufacturing, engineering) wait for proven results | Industry and market scope (4.1.3) |
|  | Market scope | Q10, Q11 | F3: firms expanding internationally must activate AI use; F14: internationally active firms adopt faster to unify cross-branch policy | Industry and market scope (4.1.3) |
|  | Regulatory environment | Q10, Q11 | F7, F13, F14: absence of clear regulation increases risk and hesitation | Regulatory environment (4.1.3) |
| *Emergent (inductive)* | *Resistance to change* | Q2, Q3 emerged inductively across responses | F3: "There are some concerns about losing jobs and career opportunities which may lead to resistance to AI adoption" | Resistance to change (4.1.4, Table 3) |
|  | *Public awareness* | Q2, Q3, Q5 emerged inductively across responses | F1, F3, F8, F10, F13, F14: limited awareness results in hesitation and delayed implementation | Public awareness (4.1.4, Table 3) |
